# Osteopontin Involves Cisplatin Resistance and Poor Prognosis in Oral Squamous Cell Carcinoma

**DOI:** 10.1155/2015/508587

**Published:** 2015-09-30

**Authors:** Sheng-Dean Luo, Yi-Ju Chen, Chien-Ting Liu, Kun-Ming Rau, Yi-Ching Chen, Hsin-Ting Tsai, Chang-Han Chen, Tai-Jan Chiu

**Affiliations:** ^1^Department of Otolaryngology, Kaohsiung Chang Gung Memorial Hospital, Kaohsiung 83301, Taiwan; ^2^Chang Gung University College of Medicine, Kaohsiung 83301, Taiwan; ^3^Kaohsiung Chang Gung Head and Neck Oncology Group, Cancer Center, Kaohsiung Chang Gung Memorial Hospital, Kaohsiung 83301, Taiwan; ^4^Institute of Clinical Medical Sciences, Chang Gung University, Kaohsiung 83301, Taiwan; ^5^Department of Anatomic Pathology, E-Da hospital, I-Shou University, Kaohsiung 83301, Taiwan; ^6^Division of Hematology-Oncology, Department of Internal Medicine, Kaohsiung Chang Gung Memorial Hospital, Kaohsiung 83301, Taiwan; ^7^Center for Translational Research in Biomedical Sciences, Kaohsiung Chang Gung Memorial Hospital, Kaohsiung 83301, Taiwan; ^8^Department of Applied Chemistry and Graduate Institute of Biomedicine and Biomedical Technology, National Chi Nan University, Nantao 54561, Taiwan

## Abstract

*Background.* Osteopontin (OPN) is a multifunctional cytokine involved in cell survival, migration, and adhesion. However, its role in chemosensitivity in locally advanced oral squamous cell carcinoma (OSCC) in humans has not yet been investigated. *Methods.* We enrolled 121 patients with locally advanced stage IVA/B OSCC receiving cisplatin-based IC followed by CCRT from January 1, 2006, through January 1, 2012. Immunohistochemistry was used to assess OPN expression in OSCC patients' biopsy specimens from paraffin blocks before treatment. In addition, MTT/colony formation assay was used to estimate the influence of OPN in an oral cancer cell line treated with cisplatin. *Results.* Of the 121 patients, 94 had positive OPN findings and 52 responded to IC followed by CCRT. Positive osteopontin immunostaining also correlated significantly with positive N status/TNM stage/male gender and smoking. Univariate analyses showed that patients whose tumors had a low expression of OPN were more likely to respond to chemotherapy and have a significantly better OS than those whose tumors had a high expression of OPN. Multivariate analysis revealed that prolonged survival was independently predicted for patients with stage IVA disease, negative lymph nodes, and negative expressions of OPN and for those who received chemotherapy with Docetaxel/cisplatin/fluorouracil (TPF). An oral cancer line stimulated with OPN exhibited a dose-dependent resistance to cisplatin treatment. Conversely, endogenous OPN depletion by OPN-mediated shRNA increased sensitivity to cisplatin. *Conclusions.* A positive expression of OPN predicts a poor response and survival in patients with locally advanced stage IVA/B OSCC treated with cisplatin-based IC followed by CCRT.

## 1. Background

Oral squamous cell carcinoma (OSCC) constitutes a major proportion of head and neck squamous cell carcinoma in the Taiwan and South-East Asia [1]. In Taiwan, two-thirds of the patients with this disease initially present with locally advanced disease [2] and OSCC rates are fourth among cancer-related deaths [1] among middle-aged male patients [3]. Despite advances in multidisciplinary treatment modalities, no improvement in the 5-year survival rate has been achieved over the past 20 years [4]. The standard treatment for OSCC remains radical resection whenever feasible and concurrent chemoradiotherapy (CCRT) when the tumor is unresectable [5]. Unfortunately, the prognosis of unresectable OSCC treated with a nonsurgical approach is poor, median survival ranging from 2 to 12 months [6–8]. Recently, cisplatin-based induction chemotherapy (IC) with cisplatin/fluorouracil (PF) or docetaxel/cisplatin/fluorouracil (TPF) has been reported to improve 5-year survival rates in patients with locally advanced disease [9–12]. In addition, according to one important cancer review study, cisplatin is the mainstay adjunctive chemotherapeutic agent used as a component of IC and CCRT in the treatment of locally advanced HNSCC [13]. Therefore, cisplatin resistance is one of the most important problems in the treatment of unresectable OSCC.

Osteopontin (OPN) is an arginine-glycine-aspartate-containing adhesive glycoprotein expressed in the kidney, macrophages, vascular smooth muscle cells, and many cells of the epithelial linings [14]. OPN is known to be involved in bone resorption, wound repair, immune function, angiogenesis, cell survival, and cancer biology [15] and is particularly strongly associated with tumorigenesis. It is expressed in various cancer cells found in breast cancer, gastric cancer, lung cancer, and oral cancer [1, 16–18]. In one previous oral cancer study, patients with high tumor expression of OPN were found to be more likely to have a poor prognosis [1]. OPN has recently been reported to induce resistance to chemotherapy in mouse breast cancer and non-small cell lung cancer cells [19, 20]. However, its role in the development of cisplatin resistance in human oral cancer is not known.

Therefore, the purpose of this study was to evaluate whether OPN expression can affect the treatment response and survival in patients with OSCC treated with cisplatin-based IC followed by CCRT. The role OPN might play in cisplatin's effect on one oral cancer cell line was also investigated.

## 2. Methods

### 2.1. Patients and Treatment

A total of 121 patients with pathologically proven locally advanced stage IVA/B OSCC were treated with IC followed by CCRT between January 1, 2006, and January 1, 2012, at Kaohsiung Chang Gung Medical Center (Taiwan). To be included, all the patients had to have a biopsy-proven nonmetastatic IV (M0) oral squamous cell carcinoma, have no synchronous primary tumors, and be ≥18 years old. In addition, the patients had to have a performance status (PS) of ≤2 on the Eastern Cooperative Oncology Group (ECOG) scale, adequate bone marrow, hepatic and renal function (creatinine clearance >60 mL/min), and a computed tomography or magnetic resonance image scan of the head and neck region within three weeks prior to the initiation of treatment. Clinicopathological information including age, gender, tumor (T) stage, nodal (N) status, TNM stage, and survival was obtained from the patients' clinical records. The histories of betel nut chewing, alcohol drinking and tobacco use were obtained by oral interview and detailed questioning during the patients' first visit to the otolaryngology clinic of the hospital. The IC consisted of cisplatin 75 mg/m^2^ and fluorouracil (5-FU) (1000 mg/m^2^) given as a continuous 24 h infusion for four days or docetaxel 60 mg/m^2^, cisplatin 75 mg/m^2^, and fluorouracil 600 mg/m^2^/day continuous 24 h infusion for four days. After IC, all patients received CCRT. During the CCRT, cisplatin was administered weekly at a dose of 40 mg/m^2^. RT was delivered 6-7 weeks after the completion of the IC with a linear accelerator. The response to IC followed by CCRT was assessed according to the World Health Organization (WHO) criteria. Surgery was performed six to twelve weeks after completion of IC followed by CCRT regimen for patients who had residual disease. Surgery was also allowed for patients who did not complete chemoradiation and had resectable residual disease at the primary site or in the neck. Patients were evaluated by CT scan or MRI of the head and neck every three months. The protocol for this study was approved by the Institutional Review Board of Chang Gung Medical Center (Taiwan).

### 2.2. Immunohistochemical Staining for OPN

Representative formalin-fixed, paraffin-embedded tissue blocks were retrieved and sectioned for the IHC study. The sections were deparaffinized, treated with 3% hydrogen peroxide for 10 min, and then microwaved in 10 mM citrate buffer (pH 6.0) to unmask the epitopes. The sections were then incubated with diluted OPN antibody (AKm2A1, 1 : 100) for 1 hr. After washing, horseradish peroxidase/Fab polymer conjugate (PicTure-Plus kit; Zymed, South San Francisco, CA) was applied to the sections for 30 min. Finally, the sections were incubated with diaminobenzidine for 5 min to develop the signals. A negative control study was simultaneously performed by omitting the primary antibody. All sections were evaluated by a pathologist, unaware of the clinical data. In the assessment of OPN expression, the sections were examined under a microscope at 200x magnification. Positive OPN immunostaining was defined as detectable immunoreactivity in the perinuclear and/or other cytoplasmic regions in at least 10% of the cancer cells. To evaluate expression of OPN, 10 fields (within the tumor and at the invasive front) were selected, and expression in 1000 tumor cells (100 cells/field) was evaluated using high-power (×400) microscopy [21].

### 2.3. Cell Culture and Transfection

Human SAS cells were obtained from American Type Culture Collection, cultured in DMEM with 10% FBS, 1% (v/v) penicillin-streptomycin solution, and maintained at 37°C in 5% CO_2_ humidified air. SAS cells (5 × 10^4^) were seeded into 6-well dishes and cultured at 37°C in 5% CO_2_ humidified air. After 24 hours, shOPN and shControl plasmids were transfected into the cells with lipofectamine reagent according to manufacturer's instructions, followed by further incubation for 24 hours at 37°C in 5% CO_2_. SAS cells stably expressing target genes were selected with 450 *μ*g/mL G418 for two weeks (Calbiochem Novabiochem, San Diego, CA, USA). The cells were then harvested for western blotting.

### 2.4. Western Blotting Assay

Cell pellets were lysed in RIPA lysis buffers (1 mM Na_3_VO_4_, 25 mM NaF, and 1x protease inhibitor cocktail protease inhibitor cocktail). Protein concentrations were determined by spectrophotometry. Sample was electrophoresed by 10% SDS-PAGE gel and transferred to PVDF membranes. The membranes were then blocked with 5% nonfat dry milk for 1 h at room temperature and incubated with primary antibodies. Monoclonal anti-antibodies, OPN and *β*-actin, were purchased from Santa Cruz and incubated with membrane at room temperature for 1 hour. HRP-conjugated secondary antibody was incubated at room temperature for 1 hour. The membrane was then developed using an enhanced chemiluminescence system and exposed to X-ray film.

### 2.5. Cell Viability Assay and Colony Formation Assay

Cells were plated onto 6 wells at 1 × 10^5^ cells/well and transfected with shControl or shOPN plasmids before incubation overnight. The next day, the cells were treated with 0 or 20 *μ*M of cisplatin for 72 hours, and the cell number was counted after using trypan Blue. For colony formation assay, cells were seeded in 60 mm dishes at a density of 1,000 cells and cultured for 21 days at 37°C under 5% CO_2_. Colonies were counted after staining with 0.01% crystal violet. All growth experiments were carried out in triplicate.

### 2.6. Drug Treatment

Cells were treated for the indicated time with chemotherapeutic drug (cisplatin) at the concentration for assay of cell survival.

### 2.7. Statistical Analysis

Statistical analyses of 2 × 2 tables of categorical variables were performed using Pearson's *x* ^2^ test or Fisher's exact test, where appropriate. Survival probability analyses were performed using the Kaplan-Meier method. Survival was calculated from the date of start of chemotherapy to the date of death or most recent follow-up. Progression-free survival (PFS) was defined as the time from the date of first chemotherapy to the date of first observation of disease progression, or relapse, or death due to any cause. Significance between group differences was assessed by log-rank test. Multivariate analyses were performed using a logistic regression model for response and Cox regression models for PFS and overall survival (OS). Factors with *p* values < 0.05 in univariate analyses were examined in multivariate regression models. All statistical tests were two-sided, with significance defined as *p* < 0.05. All statistical operations were performed using SPSS version 13.

## 3. Results

### 3.1. Patient Characteristics

We enrolled 121 eligible patients (117 males; 96.7%) from January 2006 to January 2012. The median age was 50.4 years (range 32–72 years). Thirty-five patients had IVa disease and 86 had stage IVb. All patients completed IC and received follow-up CCRT. Eighty-one (66.9%) patients received IC with PF and 40 received IC with TPF.

### 3.2. OPN Expression in OSSC Patients

**Figure 1:**
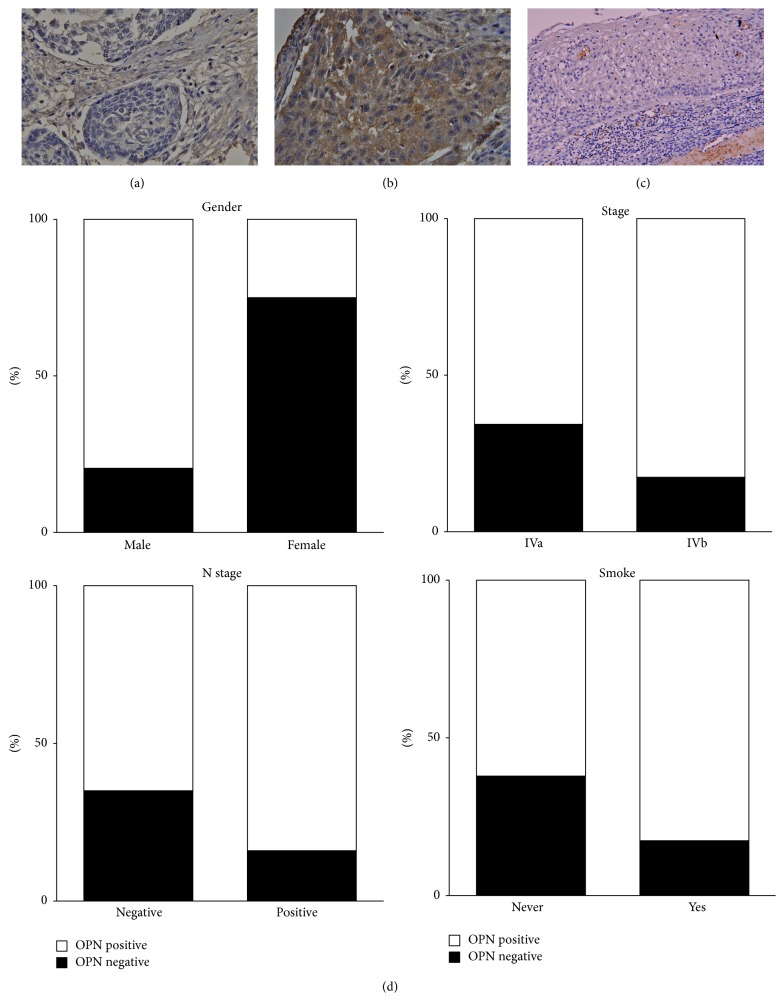
Immunostaining for OPN expression in OSCC patient samples. (a) The representative case of OSCC shows absence of OPN immunoreactivity (400x magnification). (b) The representative case of OSCC shows positive of OPN immunoreactivity in the cytoplasm of the neoplastic squamous epithelial cells (200x magnification). (c) Adjacent noncancerous tissue showed no OPN expression. (d) Statistical analysis showed that OPN expression levels in OSCC sample significantly correlated with gender, stage, N stage, and smoking.

Before treatment, tumor OPN expression levels were assessed in all patients. To investigate whether there was a positive expression of OPN associated with various prognostic factors, such as age, gender, and TNM pathologic classification, we classified the patients into two groups based on their immunohistochemical results (negative* versus* positive OPN expression) (Figures 1(a) and 1(b)). In addition, immunohistochemistry found OPN was not expressed in noncancerous tissue (Figure 1(c)). Positive OPN immunostaining in the OSCC was mainly present in the cytoplasm of the neoplastic squamous epithelial cells. Ninety-four patients were found positive for OPN expression, and 27 were not. As can be seen in Table 1, a summary of the results of the OPN immunostaining of the cancer cells and its correlation with the clinicopathologic variables, the patients in the positive and negative OPN expression groups did not significantly differ in age, T stage, alcohol drinking, or betel nut chewing (Table 1). Positive OPN expression was associated with male gender (*p* = 0.034), positive lymph node status (*p* = 0.019), TMN stage IVB (*p* = 0.044), and cigarette smoking (*p* = 0.021). As shown in Figure 1(d), a summary of the results of our statistical analysis, multivariate analysis revealed that patients of male gender and patients with positive lymph node status and TMN stage IVb had positive expressions of OPN.

### 3.3. Clinicopathological Factors, OPN Expression, and Treatment Response

The overall response rate after CCRT for all patients was 43.0%, 52/121 with 24 complete responses and 28/121 with partial responses. Thirty-four had stable disease and 35 had progressive disease. In our univariate analyses, patients with negative expression of OPN had a higher treatment response (19/27, 70.4%) than the positive expression group (33/94, 50%) (*p* = 0.002, Table 2). In addition, patients with stage IVa disease, with negative lymph node metastases and without cigarette smoking habits, had better treatment response (CR and PR). Interestingly, in our multivariate analyses, patients with negative node status, TMN stage IVa disease, and negative OPN expressions were also found to have a good response to treatment.

### 3.4. Clinicopathological Factors, OPN Expression, and Patient Outcome

**Figure 2:**
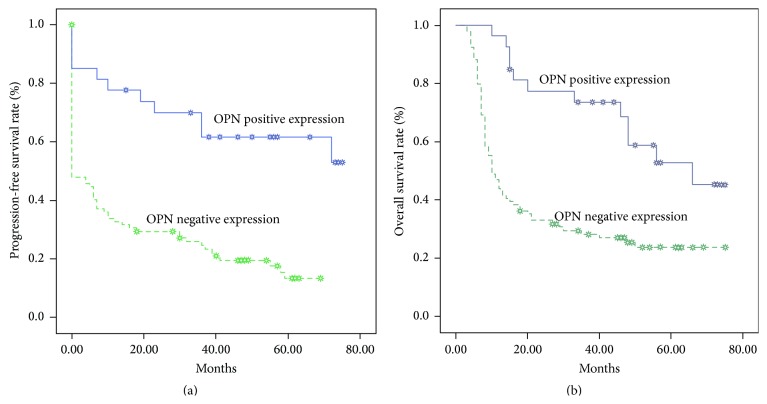
Kaplan-Meier estimates of the probability of survival. (a) Progression-free survival (PFS) and OPN expression. (b) Overall survival (OS) and OPN expression.

The median follow-up was 27.3 months (3–75 months). The 5-year PFS rate was 24.2%, and the 5-year OS rate was 30.2%. The 5-year PFS for patients with negative expression of OPN was 61.8%, compared with 13.2% for patients with positive expression of OPN (*p* < 0.01, Figure 2(a)). The 5-year OS rate was significantly higher in patients with negative expression of OPN (50.3%) than in those with positive expression of OPN (23.7%) (*p* < 0.001, Figure 2(b)). Univariate analysis showed that TNM stage, lymph node status, cigarette smoking, and OPN were important factors affecting the PFS and OS (Table 3). Multivariate analysis based on a Cox regression model found OPN expression, lymph node status, and TMN status to the prognostic factors in PFS and OS (Table 4).

### 3.5. OPN Promoted OSCC Cell Proliferation

**Figure 3:**
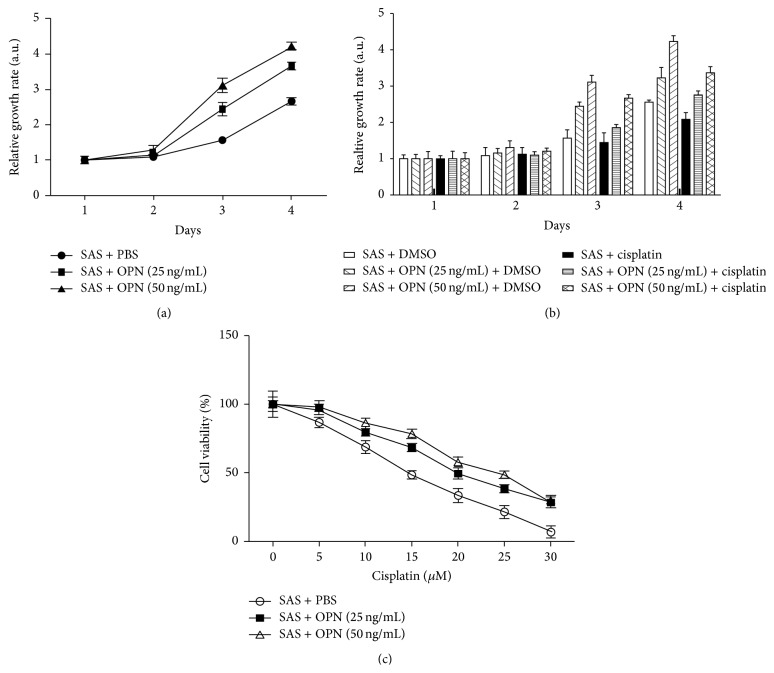
OPN promoted cell proliferation and drove cisplatin resistance in an oral cancer cell line. (a) SAS cells stimulated with OPN protein promoted cell growth. SAS cells were treated with indicated concentrations of OPN, and cell growth was analyzed on days 1–4 by MTT assay. Data were normalized against the OD_570_ value on day 1 of each treatment. The results represent the mean ± SD of three independent experiments. (b) OPN affected the chemosensitivity of OSCC cells to cisplatin. SAS cells were cultured in the presence or absence cisplatin and/or OPN in a dose-dependent manner, and their viability was measured. (c) OPN stimulated SAS cells were incubated with increasing concentrations of cisplatin for 72 hours, and their viability was measured and was expressed in percentage.

To elucidate the role of OPN in cell proliferation, recombinant human OPN was executed to SAS cells (human tongue carcinoma cell line) to determine if increased OPN protein could confer a proliferative advantage to SAS cells. The proliferation rate was significantly increased in matricellular-OPN in a dose-dependent manner in SAS cells (Figure 3(a)). This result demonstrates that one of the major roles of OPN is to promote growth of OSCC cells.

### 3.6. Cells Stimulated with OPN Resistance to Cisplatin

To gain further insight into the biological effect that OPN might have on chemoresistance to cisplatin, we performed MTT assay to assess cell viability in cells incubated with OPN and/or cisplatin treatment. As shown in Figure 3(b), parental SAS cells were found to be significantly more sensitive to cisplatin than the cells obtained from DMSO control group. Notably, in the presence of cisplatin, SAS cells stimulated with higher doses of OPN exhibited a lower sensitivity to the cytotoxic effects of cisplatin (Figure 3(b)), suggesting that OPN affected the efficacy of cisplatin in the treatment of SAS. Next, we examined the cell viability in SAS cells stimulated with OPN under cisplatin treatment at various doses for 72 hours. As can be seen in Figure 3(c), SAS cells treated with high dose of OPN were more viable than the cells treated with vehicle or lower doses of OPN (Figure 3(c)). These results indicate that OPN affects the efficacy of cisplatin in the treatment of this oral cancer cell line.

### 3.7. Endogenous OPN Depletion by siRNA Sensitizes Cytotoxicity to Cisplatin in Oral Cancer Cell Line

**Figure 4:**
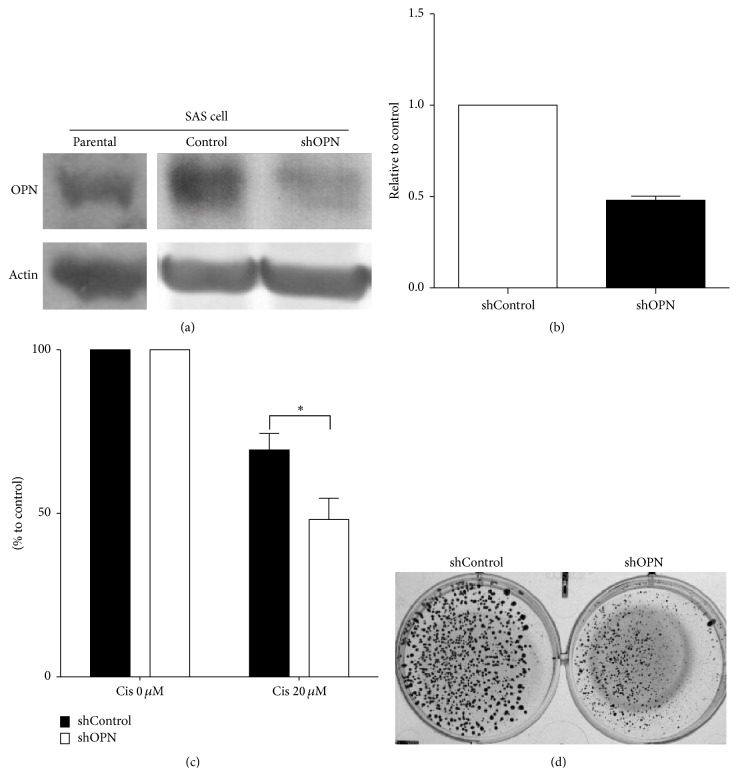
Endogenous OPN depletion by OPN shRNA sensitized the cytotoxicity to cisplatin in head and neck cancer cells. (a and b) The endogenous expression level of OPN was determined by western blotting and quantitative RT-PCR in SAS cell transfected with shControl or shOPN. Data are representative of three independent experiments. (c) SAS cell transfected with shControl or shOPN were incubated with 20 *μ*M cisplatin for 72 hours, and their viability was measured and compared to that of untreated respective cells. (d) The colony formation ability of shControl and shOPN cells treated with cisplatin (20 *μ*M).

The expression level of OPN was determined by western blotting and Q-RT-PCR in SAS cell transiently transfected with shControl or shOPN (Figures 4(a) and 4(b)). FaDu cell transfected with shControl or shOPN cells were incubated with 20 *μ*M of cisplatin for 72 hours. The viability of cells transfected with shOPN was significantly decreased compared to cells transfected with shControl (Figure 4(c)). Colony formation assay of cells transfected with shControl or shOPN and cultured in the presence of cisplatin (20 *μ*M) revealed a decrease in the colony formation ability of cell transfected with shOPN compared to shControl (Figure 4(d)). Together, these results demonstrate that OPN expression influences the response of this oral cancer cell line to cisplatin treatment.

## 4. Discussion

In this study, we wanted to find out if there was a correlation between OPN expression and treatment response and clinical outcomes in OSCC patients and we performed experiments to determine whether OPN might play an important role in cisplatin resistance in an oral cancer cell line. OPN was positively expressed in 78 percent of our patients with stage IVA/B unresectable OSCC. Patients with this positive expression had a lower treatment response rate to IC followed by CCRT than those with negative expression of OPN (*p* = 0.002; Table 2). Negative OPN expression was associated with significantly longer PFS and OS (*p* < 0.001, resp.; Tables 3 and 4). Positive expression of OPN was independently associated with a high risk of cancer death (HR 2.206, *p* = 0.028) and recurrence of cancer (HR 2.509, *p* = 0.009) in our multivariate analysis. These findings suggest OPN might be used as both a prognostic biomarker and a predictive marker in unresectable OSCC.

OPN (Osteopontin), also known as SPP1, is a member of the small integrin-binding ligand N-linked glycoprotein (SIBLING) family and is secreted in mammalian cells. Secreted OPN has been discovered in tissue matrix, including bone, fibroblast, osteocyte, dendritic cells, macrophages, and activated T cells [22]. This study found immunohistochemical expression of OPN protein to be weakly positive in the stromal cells. However, the role of stromal-derived OPN is still not well understood. Avirović et al., studying the relationship between stromal OPN expression and clinicopathological parameters and clinical outcomes, found no significant association [23]. Tumor OPN plays an important role in tumor development, particularly in tumor invasion and metastasis. The overexpression of OPN in tumor tissues has been associated with a worse prognosis in a variety of malignancies, including cancers of the breast, stomach, lung, esophagus, and OSCC [24]. OPN can interact with two types of receptors: integrin, including a4b1, a9b1, a9b4, av(b1, b3, b5), and CD44, which in turn can activate many different signal transduction pathways to regulate cell growth [25–27]. It is now well accepted that OPN is a promising target for therapeutic drugs in cancer therapy.

Only few studies, however, have addressed the role of OPN in chemotherapy resistance, though one previous report has shown pretreatment low plasma OPN expression led to better treatment response and longer survival for locally advanced HNSCC patients treated by CCRT [28]. Our current study found patients whose tumors had a low expression of OPN to more likely respond to chemotherapy and have a significantly better OS than those whose tumors had highly expressed OPN. For further confirmation of correlation of OPN and cisplatin resistance, we also tried to add secretory OPN to culture media of an oral cancer cell line and knock-down OPN in SAS to study the involvement of OPN in cisplatin resistance. Our findings were consistent with a previous report by Graessmann et al., which showed an association between secreted OPN and medium-induced chemoresistance of mouse breast cancer cells, suggesting that extracellular OPN may mediate antiapoptotic signaling [19]. In addition, resent study demonstrated that OPN increased chemo-resistance to cisplatin in SBC-3 cells by suppressing bcl-2 protein down-regulation, thereby blocking the caspase-9- and caspase-3-dependent cell apoptosis [20].

Betel nut chewing, cigarette smoking, and alcohol consumption are important risk factors for the development of OSCC in South-East Asia and Taiwan, where they represent major threats to public health. It is of special interest that in our study specimens obtained from our patients who with habitually smoked cigarettes predominantly had a positive expression of OPN. Cigarette smoking has a long-standing association with cardiovascular disease and there is a wealth of evidence concerning the effects of smoking on key pathological processes such as vascular injury, vascular dysfunction, inflammation, and thrombosis [29]. Recent studies have shown an emerging and widespread role of OPN in inflammation, tissue remodeling, and vascular disease [30]. In addition, some* in vitro* and* in vivo* studies have indicated that OPN is potentially involved in the pathogenesis of smoking-related obstructive and interstitial lung disease [31]. In previous reports, overexpression of OPN was found to be involved in OSCC. In the current investigation, we found a significant correlation between smoking and positive expression OPN in the tissue samples obtained from patients who habitually smoked cigarettes. This finding awaits confirmation by prospective studies with large numbers of patients.

Prognosis for nonmetastatic stage IV OSCC patients is dismal, though it has been found that IC followed by CCRT can prolong survival. Resistance to chemotherapy is always a major problem in progressive cancer, when tumor cells begin to amplify their proliferation, metastasis, and invasion to distant organs, leading to poor outcome and low survival [32]. Cancer treatment with chemotherapeutic agents largely depends on activation of apoptosis programs. The failure to undergo apoptosis in response to anticancer therapy may result in cancer resistance [33]. Although the mechanism underlying cisplatin resistance in OSCC remains unclear, there are some possible explanations. First, it has been observed that multiple drug resistant (MDR) proteins are involved in the chemoresistance in variety of cancers [34]. Classic MDR is caused mainly by the overexpression of multidrug resistance gene (MDR1) encoding the P-glycoprotein (P-gp), which is thought to act as an energy-dependent drug efflux pump [35]. P-gp expression in tumors may increase drug resistance and impair patients' response to chemotherapy [36]. OPN uses a conserved arginine-glycine-aspartic acid (RGD) domain to bind to multiple integrin receptors and trigger cell signaling and promote cell adhesion, migration, and flattening [37]. OPN could upregulate P-gp expression, which is present in PC-3 cell membrane and cytoplasm, and also increase P-gp expression in DU145 prostate cancer cells [38]. Second, in the head and neck cancer, the positive expression of osteopontin has been associated with an increase in Aurora-A expression [39] and, in esophageal squamous cell carcinoma cells, overexpression of Aurora-A inhibits the cisplatin- or UV irradiation-induced apoptosis.

It is of special interest in the current study that we did not find IC with TPF followed by CCRT to have a better response than PF followed by CCRT in OSCC. Moreover, we did not find stage IVa/b OSCC patients receiving TFP to have a better PFS and OS than those receiving PF. In locally advanced disease groups of patients, IC is known to improve response rates, quickly reduce symptoms, possibly predict subsequent radioresponsiveness, and lead to a reduced rate of distant metastases [40]. The chemotherapeutic regime most often used to treat these patients is cisplatin and infusional 5FU. Results from randomized TAX-323 and TAX-324 studies have shown that the addition of docetaxel to IC with cisplatin and 5-fluorouracil (5-FU) (PF) improves survival in patients with locally advanced HNSCC [41, 42]. Although findings from these randomized controlled trials have shown a 14% increase in 3-year overall survival beyond platinum-based doublet chemotherapy regimens, the proportion of oral cancer patients in those studies was <15%.

Our study is limited in that only a small number of our patients received TPF. Furthermore, studies of a more homogeneous, larger number of patients may be needed to validate our findings.

## 5. Conclusion

In conclusion, this study found that the positive expression of OPN predicts a poor response and survival to cisplatin-based IC followed by CCRT in patients with locally advanced stage IVa/b OSCC. Thus, we conclude that OPN mediated cisplatin resistance contributes to a poorer clinical outcome in patients with locally advanced inoperable OSCC treated with cisplatin-based IC and CCRT. In this context, it is strongly recommended that tissue be collected to assess OPN expression before cisplatin-based IC and CCRT are started. If patients with lymph node metastases and advanced tumors may have higher change of positive OPN expression, then other treatment modalities may be considered.

## Figures and Tables

**Table 1:** Correlation between expression of OPN and clinicopathological factors of stage IVa/b OSCC.

	Number of patients	OPN		*p*	Multivariates analysis	*p*
		Negative expression	Positive expression		OR (95% CI)	
Age						
<60	102 (84.3%)	22 (21.6%)	80 (78.4%)	0.764		
≥60	19 (15.7%)	5 (26.3%)	14 (73.7%)			
Gender						
Male	117 (96.7%)	24 (20.5%)	93 (79.5%)	0.034^*∗*^	1	
Female	4 (3.3%)	3 (75%)	1 (25%)		0.067 (0.06–0.791)	0.032
Grade						
Well	77 (63.6%)	17 (22.1%)	60 (77.9%)			
Moderate	40 (33.1%)	10 (25%)	30 (75%)	0.517		
Poor	4 (3.3%)	0 (0%)	4 (100%)			
Stage						
Iva	35 (28.9%)	12 (34.3%)	23 (65.7%)	*0.044* ^*∗*^	1	
IVb	86 (71.1%)	15 (17.4%)	71 (82.6%)		2.755 (1.042–7.279)	0.041
T stage						
1/2	28 (10.5%)	5 (17.9%)	23 (82.1%)	0.612		
3/4	93 (89.5%)	22 (23.7%)	71 (76.3%)			
N stage						
Negative	40 (21.1%)	14 (35.0%)	26 (65.0%)	*0.019* ^*∗*^	1	
Positive	81 (78.9%)	13 (16.0%)	68 (84.0%)		3.534 (1.363–9.164)	0.009
Alcohol drinking						
Never	30 (24.8%)	7 (23.3%)	23 (76.3%)	0.877		
Yes	91 (75.2%)	20 (22.0%)	71 (78.0%)			
Smoking						
Never	29 (24.0%)	11 (37.9%)	18 (62.1%)	*0.021* ^*∗*^		
Yes	92 (76.0%)	16 (17.4%)	76 (82.6%)			
Betel nuts						
Never	31 (25.6%)	10 (32.3%)	21 (67.7%)	0.123		
Yes	90 (74.4%)	17 (18.9%)	73 (81.1%)			

OR, odds ratio; CI, confidence interval.

^*∗*^Indicates significantly difference (*p* < 0.05).

**Table 2:** Relationship between treatment response and clinicopathological factors.

	Treatment response			Multivariates	
	SD/PD	CR/PR	*p*	OR (95% CI)	*p*
Age					
<60	58 (56.9%)	44 (43.1%)	1.000		
≥60	11 (57.9%)	8 (22.1%)			
Gender					
Male	67 (57.3%)	50 (42.7%)	1.000		
Female	2 (50%)	2 (50%)			
Grade					
Well	41 (53.2%)	36 (46.8%)			
Moderate	24 (60%)	16 (40%)	0.165		
Poor	4 (100%)	0 (0%)			
Stage					
IVa	13 (37.1%)	22 (62.9%)	*0.005* ^*∗*^	1	
IVb	56 (65.1%)	30 (34.9%)		0.310 (0.128–0.752)	*0.010*
T stage					
1/2	15 (53.6%)	13 (46.4%)	0.674		
3/4	54 (58.1%)	39 (41.9%)			
N stage					
Negative	16 (40.0%)	24 (60.0%)	*0.008* ^*∗*^	1	
Positive	53 (65.4%)	28 (34.6%)		0.354 (0.151–0.834)	*0.017*
Chemotherapy					
PF	51 (63%)	30 (37%)	0.147		
TPF	18 (45%)	22 (55%)			
Alcohol drinking					
Never	17 (56.7%)	13 (43.3%)	0.964		
Yes	52 (57.1%)	39 (42.9%)			
Smoking					
Never	11 (37.9%)	18 (62.1%)	*0.017* ^*∗*^		
Yes	58 (63.0%)	34 (37.0%)			
Betel nuts					
Never	16 (51.6%)	15 (48.4%)	0.480		
Yes	53 (58.9%)	37 (41.1%)			
OPN					
Positive	8 (29.6%)	19 (70.4%)	*0.002* ^*∗*^	1	
Negative	61 (64.9%)	33 (35.1%)		0.320 (0.120–0.854)	*0.023*

CR, complete response; PR, partial response; SD, stable disease; PD, disease progression; PF, cisplatin/fluorouracil; TPF, docetaxel/cisplatin/fluorouracil; OR, odds ratio; CI, confidence interval.

^*∗*^Indicates significantly difference (*p* < 0.05).

**Table 3:** Correlation between the clinicopathological features and 5-year progression-free survival in oral squamous cell carcinoma.

Variables	Number of patients	Cumulative 5-year PFS rate	*p*	5-year PFS	*p*
				HR (95% CI)	
Age					
<60	102	23.2%	0.992	
≥60	19	26.3%		
Gender					
Male	117	23.2%	0.372	
Female	4	50.0%		
Stage					
IVa	35	38.7%	<0.001^**∗**^	1	
IVb	86	20.0%		2.097 (1.255–3.505)	*0.005*
T stage					
1-2	28	26.2%	0.735	
3-4	93	23.4%		
N stage					
Negative	40	47.3%	<0.001^**∗**^	1	
Positive	81	12.0%		1.940 (1.148–3.279)	0.013
Chemotherapy					
PF	83	23.0%	0.465		
TPF	38	26.4%			
Alcohol					
Never	30	33.1%	0.206	
Yes	91	20.8%		
Smoking					
Never	29	39.8%	***0.009*** ^**∗**^	
Yes	92	19.0%		
Betel nuts					
Never	31	22.7%	0.337	
Yes	90	23.5%		
OPN					
Negative	27	61.8%	<0.001^**∗**^	1	
Positive	94	13.2%		2.509 (1.256–5.009)	*0.009*

CI, confidence interval; HR, hazard ratio.

^*∗*^Indicates significantly difference (*p* < 0.05).

**Table 4:** Correlation between the clinicopathological features and 5-year overall survival in oral squamous cell carcinoma.

Variables	Number of patients	Cumulative 5-year overall survival rate	*p*	HR (95% CI)	*p*
Age					
<60	102	31.0%	0.628	
≥60	19	26.3%		
Gender					
Male	117	29.4%	0.415	
Female	4	50.0%		
Stage					
IVa	35	64.5%	***<0.001***	1	
IVb	86	15.3%		3.936 (2.129–7.277)	*<0.001*
T stage					
1-2	28	38.7%	0.488	
3-4	93	27.4%		
N stage					
Negative	40	51.5%	***0.002***	1	
Positive	81	20.5%		2.436 (1.422–4.175)	*0.001*
Chemotherapy					
PF	83	24.7%	0.123	
TPF	38	41.0%		
Alcohol					
Never	30	33.3%	0.538	
Yes	91	29.1%		
Smoking					
Never	29	48.0%	***0.010***	1	
Yes	92	24.4%		1.869 (1.014–3.355)	0.036
Betel nuts					
Never	31	33.0%	0.096	
Yes	90	29.2%		
OPN					
Negative	27	53.0%	***<0.001*** ^**∗**^	1	
Positive	94	23.7%		2.036 (1.080–3.839)	*0.028*

CI, confidence interval; HR, hazard ratio.

^*∗*^Indicates significantly difference (*p* < 0.05).
